# Hemoglobin A1c-systolic blood pressure index as a novel predictor of cardiovascular disease: evidence from three prospective cohorts

**DOI:** 10.3389/fendo.2026.1718936

**Published:** 2026-02-18

**Authors:** Ruiqi Zhang, Xuelian Chen, Benjun Zhou, Boyang Xiang, Shushu Zhu

**Affiliations:** 1 Department of Radiology, Kunshan First People’s Hospital Affiliated to Jiangsu University, Kunshan, China; 2 Department of Cardiology, The Second Affiliated Hospital of Nanjing Medical University, Nanjing, China

**Keywords:** cardiovascular disease, diabetes mellitus, hemoglobin A1c, hypertension, prediction, systolic blood pressure

## Abstract

**Background:**

Hypertension and diabetes are major drivers of cardiovascular disease (CVD), and their coexistence confers excess risk. This study aimed to develop a hemoglobin A1c (HbA1c)-systolic blood pressure (SBP) index (HSI) to simultaneously capture glucose and blood pressure status and investigate the associations of baseline and cumulative HSI with incident CVD.

**Methods:**

Data were drawn from three population-based cohorts: the China Health and Retirement Longitudinal Study (CHARLS), the English Longitudinal Study of Ageing (ELSA), and the US Health and Retirement Study (HRS). Baseline HSI was calculated as HbA1c (%) × SBP (mmHg)/100. Cumulative HSI was derived from repeated measurements weighted by time intervals. Cause-specific Cox proportional hazards models were used to investigate linear associations of baseline and cumulative HSI with incident CVD. Additionally, restricted cubic splines were used to assess nonlinear relationships.

**Results:**

A total of 6,822 participants from CHARLS, 3,640 from ELSA, and 5,709 from HRS were included, with median follow-up of 9.0, 10.0, and 12.3 years, respectively. Across all three cohorts, the combination of elevated HbA1c and SBP was associated with the highest CVD risk. Higher baseline HSI were significantly associated with increased risks of CVD in the CHARLS (hazard ratio [HR] per 1 standard deviation [SD] increase =1.16, 95% confidence interval [CI] 1.11–1.22), ELSA (1.13, 95% CI 1.06–1.21), and HRS (1.14, 95% CI 1.10–1.19). Cumulative HSI levels were also significantly associated with elevated CVD risk (CHARLS: HR per 1 SD increase = 1.19, 95% CI 1.12–1.26; ELSA: 1.14, 95% CI 1.04–1.26; HRS, 1.15, 95% CI 1.08–1.22). No evidence of nonlinearity between baseline HSI and CVD was detected. The associations were almost consistent across demographic and clinical subgroups. The predictive performance of HSI was superior to HbA1c or SBP alone.

**Conclusions:**

HSI, a simple composite of HbA1c and SBP, was consistently associated with incident CVD across three international cohorts. Its predictive ability exceeded that of HbA1c or SBP alone, highlighting it as a pragmatic tool for integrated cardiometabolic risk assessment. The findings warrant further clinical validation.

## Graphical Abstract

**Figure:**
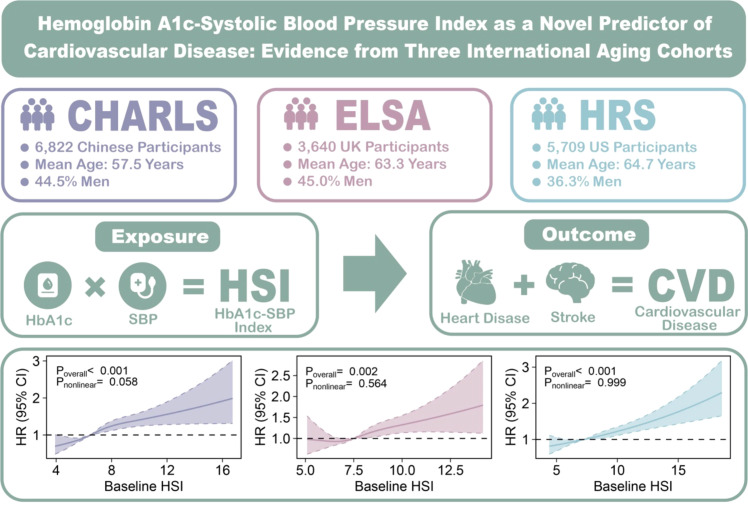

## Introduction

Cardiovascular disease (CVD) is the foremost cause of morbidity and mortality globally, with nearly 0.81 billion people affected worldwide (1). Despite substantial progress made in CVD treatment modalities, its global disease burden remains relatively high.

Hypertension and diabetes are the primary contributors to the CVD epidemic worldwide (2). The American Heart Association has identified key modifiable risk factors through its Life’s Essential 8 framework, emphasizing blood pressure and glucose control among other critical elements for cardiovascular risk reduction (3). This approach aligns with emerging understanding of cardiovascular-kidney-metabolic syndrome, highlighting the importance of metabolic factors in cardiovascular pathogenesis (4, 5).

An epidemiological study found that the prevalence of both hypertension and diabetes together in the United States nearly doubled over the past two decades, rising from 6% to 12% (6). Individuals with both hypertension and diabetes had a 197% increased risk of cardiovascular death compared with those with neither condition (6). A large observational study of 3.3 million individuals also demonstrated that hypertension and diabetes additively increased the risk of incident CVD (7). These findings highlight the urgent need for managing both hypertension and diabetes at the same time. Systolic blood pressure (SBP) and glycated hemoglobin A1c (HbA1c) represent the gold standards for assessing hypertension severity and glycemic control, respectively, with contemporary clinical practice guidelines establishing these measures as fundamental diagnostic and monitoring tools (8–11). The clinical significance of these biomarkers extends beyond their diagnostic utility, as epidemiological studies have consistently demonstrated their strong predictive value for cardiovascular outcomes (12–15). However, no existing metric simultaneously reflects the overall health status of glycemic and blood pressure control.

This study aims to develop a composite index, the HbA1c-SBP index (HSI), to quantify integrated glycemic control and blood pressure status. The longitudinal data from three cohorts in China, the UK, and the US were used to examine the associations between both baseline and cumulative index values and incident cardiovascular events, and to evaluate the predictive utility of this composite measure.

## Methods

### Study design and population

This study used data from three nationally representative prospective cohorts: the China Health and Retirement Longitudinal Study (CHARLS), English Longitudinal Study of Ageing (ELSA), and Health and Retirement Study (HRS), conducted in China, the UK, and the US, respectively. Detailed study designs are provided in the **Supplementary Methods**. The baseline data were obtained from CHARLS wave 1 (2011), ELSA wave 4 (2008–2009), and HRS wave 8 or 9 (2006 or 2008, selected based on biomarker data availability). The second survey data came from CHARLS wave 3 (2015), ELSA wave 6 (2012–2013), and HRS wave 10 or 11 (2010 or 2012, selected based on the availability of biomarker data). These two time points enabled calculation of cumulative HSI burden. Participants were subsequently followed up through the final surveys: CHARLS wave 5 (2020), ELSA wave 9 (2018–2019), and HRS wave 15 (2020) (**Supplementary Figure S1**). Ethics approval was obtained from the Ethics Review Committees of Peking University (CHARLS), London Multi-Centre Research (ELSA), and the University of Michigan (HRS), with informed consent from all participants.

**Figure 1:**
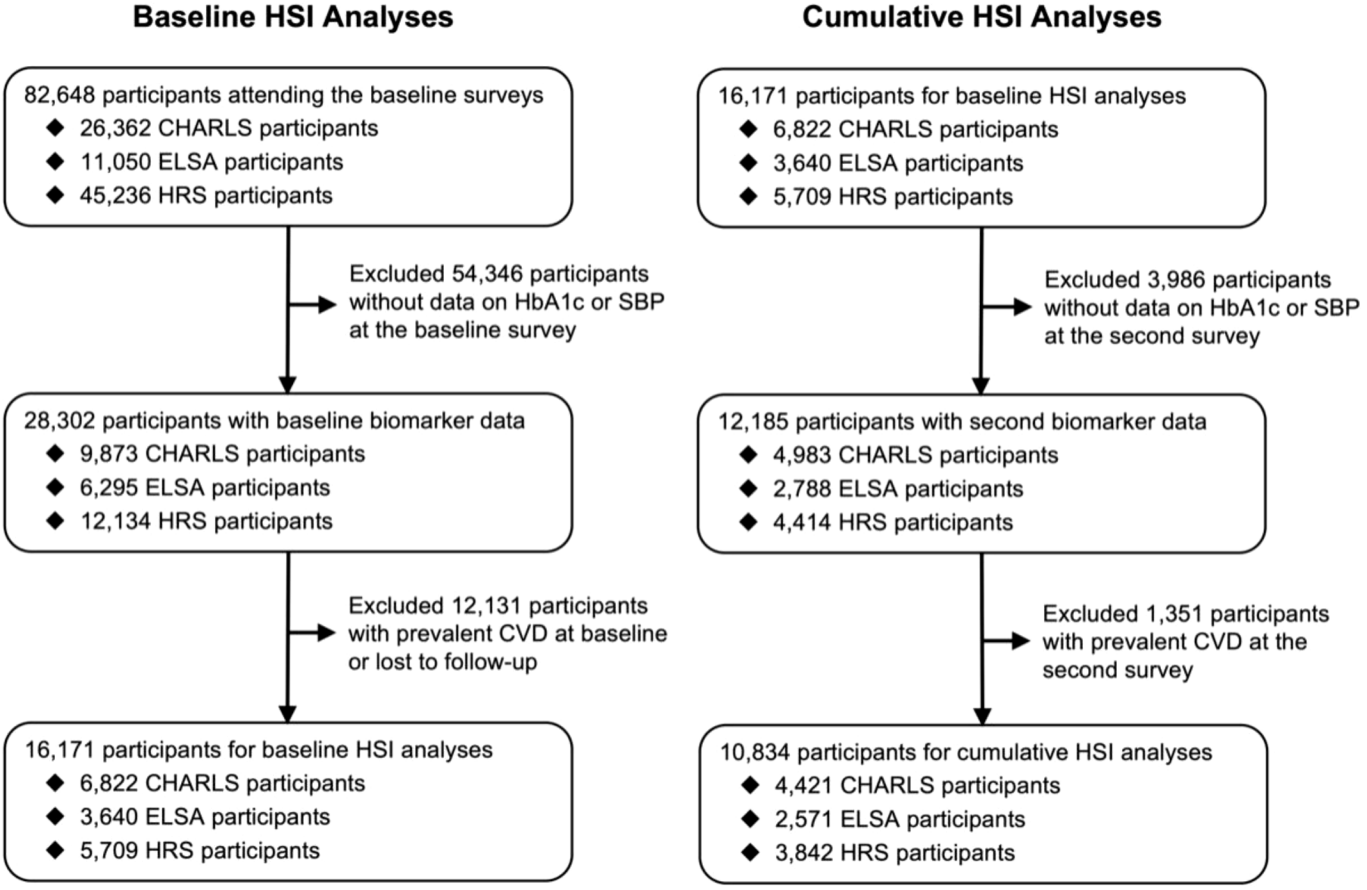
Selection flowchart of participants across three cohorts. CHARLS, China Health and Retirement Longitudinal Study; CVD, cardiovascular disease; ELSA, English Longitudinal Study of Ageing; HRS, Health and Retirement Study; HSI, hemoglobin A1c-systolic blood pressure index.

Participants with available SBP and HbA1c data at baseline were eligible for inclusion. We excluded those with established CVD at baseline or incomplete follow-up data. Participants with CVD history or those lacking SBP and HbA1c data at the second assessment were also excluded for cumulative HSI burden analyses (**Figure 1**).

### Biomarker assessment

SBP was determined by averaging two to three resting measurements (mmHg) taken within a single survey wave. HbA1c levels were measured as percentage (%) or mmol/mol units. When originally reported in mmol/mol, values were converted to percentage using the established formula: HbA1c (%) = 0.09148 × HbA1c (mmol/mol) + 2.152 (16). The calculations of baseline and cumulative HSI followed these formulas: baseline HSI = HbA1c (%) × SBP (mmHg) ÷ 100; cumulative HSI = [(baseline HSI + second HSI) ÷ 2] × time interval (years).

### Covariate assessment

The covariates were selected according to potential causal relationships, including age, sex (male or female), white ethnicity (yes or no), high school completion (yes or no), socioeconomic disadvantage (defined as a household income in the lowest quintile), current smoking status (yes or no), current alcohol intake (yes or no), non-high-density lipoprotein cholesterol (total cholesterol minus high-density lipoprotein cholesterol), body mass index (weight[kg] ÷ height[m]^2^), and current medications for hypertension, cholesterol, and diabetes (yes or no).

### Outcome ascertainment

The study outcome was incident CVD, which was identified through self-reported physician diagnoses. Participants answered standardized questions during each assessment: “Have you been told by a doctor that you have been diagnosed with heart disease, including angina, heart attack, congestive heart failure, and other heart problems?” and “Have you been told by a doctor that you have been diagnosed with stroke?” CVD diagnosis was based on affirmative responses to either question. Previously reported diagnoses were confirmed in subsequent waves, with retrospective correction of inconsistencies to ensure data integrity. The follow-up commenced from 2008–2009 for ELSA, 2011 for CHARLS, and 2008 or 2010 for HRS in baseline HSI analyses, and 2012–2013 for ELSA, 2015 for CHARLS, 2012 or 2014 for HRS in cumulative analyses. The observation continued until CVD occurrence, death, or last available survey, whichever occurred first. Non-CVD mortality was considered a competing event. The final surveys were CHARLS wave 5 (2020), ELSA wave 9 (2018–2019), and HRS wave 15 (2020).

### Statistical analyses

Participant characteristics for baseline and cumulative HSI analyses were summarized using mean (standard deviation [SD]) for continuous variables and frequency (percentage) for categorical variables. The missing covariate data were handled through multivariate imputation by chained equations using random forest methodology (17).

Cause-specific Cox proportional hazards models were employed to evaluate the relationships between HSI measures and incident CVD. The initial model (Model 0) included crude, unadjusted analysis. The second model (Model 1) incorporated fundamental demographic variables encompassing age, sex, and ethnicity. The final model (Model 2) extended beyond demographic characteristics to include a comprehensive array of socioeconomic indicators and clinical parameters, namely education levels, socioeconomic position, tobacco use, alcohol consumption, non-high-density lipoprotein cholesterol levels, body mass index, and concurrent pharmaceutical treatments for hypertension, dyslipidemia, and diabetes mellitus. Both baseline and cumulative HSI values were analyzed as continuous variables to estimate hazard ratios (HRs) with corresponding 95% confidence intervals (CIs). Additionally, HSI values were stratified into tertiles (low, moderate, high) based on the distribution within each cohort, with the lowest tertile serving as the reference category for group comparisons. The proportional hazards assumption was verified through visual inspection of scaled Schoenfeld residuals plots, and no assumption violation was found.

Cumulative hazard curves were generated to visualize CVD incidence across HSI tertiles. The log-rank tests were performed to assess statistical differences between groups. Nonlinear dose-response relationships were explored using restricted cubic splines with four knots positioned at the 5th, 35th, 65th, and 95th percentiles of HSI distribution. The optimal cutoff value of HSI for predicting cardiovascular events was determined based on the max log-rank statistic using pooled data. The applicability of the optimal cutoff value was evaluated using cumulative hazard curves.

The predictive performances of SBP, HbA1c, their additive combination, and HSI were compared by calculating Harrell’s concordance index (C-index) and estimating its 95% CI using bootstrap resampling with 1000 iterations. Moreover, 10-year receiver operating characteristic curves were also depicted.

Several sensitivity analyses were performed to ensure robustness of findings. First, subgroup analyses were conducted across demographic and clinical subgroups, and the multiplicative interaction between HSI and stratified variables was examined. Second, to mitigate potential reverse causality, we excluded participants who developed CVD within the first 2 years of follow-up and repeated the primary analyses with this restricted sample. Third, Fine-Gray subdistribution hazard models were implemented to account for the competing risk of non-CVD death, providing subdistribution HRs that more accurately reflect CVD incidence in the presence of competing events. Fourth, the follow-up time in the three cohorts was relatively inaccurate due to their study design, and we used modified Poisson regression to assess their associations to decrease the interference of inaccurate time. Fifth, HSI was reclassified using clinically meaningful cutpoints derived from established HbA1c and SBP thresholds: low risk (<6.84, corresponding to HbA1c <5.7% and SBP <120 mmHg), moderate risk (6.84–9.09), and high risk (≥9.10, corresponding to HbA1c ≥6.5% and SBP ≥140 mmHg), and the applicability of the clinical classification was assessed using cumulative hazard curves.

All statistical analyses were conducted using R software version 4.5.1 with “survival,” “rqlm,” and “cmprsk” packages. A two-sided P <0.05 indicated statistical significance.

## Results

A total of 16,171 participants were included in the baseline HSI analyses, comprising 6,822 from CHARLS (mean age [SD] 57.5 ± 9.0 years, 44.5% men), 3,640 from ELSA (mean age [SD] 63.3 ± 8.1 years, 45.0% men), and 5,709 from HRS (mean age [SD] 64.7 ± 9.5 years, 36.3% men). The median follow-up duration was 9.0, 10.0, and 12.3 years, respectively. During follow-up, 1,760 CVD events occurred in CHARLS, 937 in ELSA, and 2,282 in HRS. The cumulative HSI analyses included 10,834 participants (4,421 from CHARLS, 2,571 from ELSA, and 3,842 from HRS), with the median follow-up duration of 5.0, 6.2, and 8.3 years, respectively. The number of CVD events during follow-up was 923 in CHARLS, 448 in ELSA, and 1,117 in HRS. The baseline characteristics for both baseline and cumulative HSI analyses are presented in **Table 1**, **Supplementary Table S1**. Participants with higher baseline and cumulative HSI values were typically older and more likely to be men. They had lower educational attainment, poorer socioeconomic status, less favorable lifestyle patterns, and worse metabolic health profiles.

**Table 1:** Baseline characteristics of participants for baseline HSI analyses.

Characteristics	Baseline HSI (CHARLS)			Baseline HSI (ELSA)			Baseline HSI (HRS)		
	Tertile 1	Tertile 2	Tertile 3	Tertile 1	Tertile 2	Tertile 3	Tertile 1	Tertile 2	Tertile 3
Number	2,255	2,318	2,249	1,201	1,239	1,200	1,885	1,940	1,884
Age (years), mean (SD)	55.2 (8.5)	57.3 (8.7)	60.0 (9.1)	60.7 (7.4)	63.3 (7.9)	65.8 (8.2)	61.7 (9.5)	64.8 (9.0)	67.5 (9.0)
Men, n (%)	947 (42.0%)	1,110 (47.9%)	976 (43.4%)	500 (41.6%)	567 (45.8%)	570 (47.5%)	517 (27.4%)	760 (39.2%)	798 (42.4%)
White, n (%)	0 (0%)	0 (0%)	0 (0%)	1,177 (98.0%)	1,208 (97.5%)	1,166 (97.2%)	1,654 (87.7%)	1,604 (82.7%)	1,428 (75.8%)
High school education, n (%)	253 (11.2%)	232 (10.0%)	164 (7.3%)	971 (80.8%)	925 (74.7%)	832 (69.3%)	1,674 (88.8%)	1,648 (84.9%)	1,463 (77.7%)
Socioeconomic deprivation, n (%)	240 (10.6%)	307 (13.2%)	358 (15.9%)	171 (14.2%)	208 (16.8%)	204 (17.0%)	966 (51.2%)	1,028 (53.0%)	982 (52.1%)
Smoking, n (%)	660 (29.3%)	699 (30.2%)	679 (30.2%)	135 (11.2%)	139 (11.2%)	158 (13.2%)	257 (13.6%)	254 (13.1%)	231 (12.3%)
Alcohol consumption, n (%)	682 (30.2%)	743 (32.1%)	609 (27.1%)	1121 (93.3%)	1,159 (93.5%)	1,101 (91.8%)	775 (41.1%)	769 (39.6%)	594 (31.5%)
Hypertension, n (%)	177 (7.8%)	643 (27.7%)	1,735 (77.1%)	213 (17.7%)	536 (43.3%)	1,055 (87.9%)	665 (35.3%)	1,226 (63.2%)	1,682 (89.3%)
Diabetes, n (%)	36 (1.6%)	64 (2.8%)	367 (16.3%)	9 (0.7%)	44 (3.6%)	301 (25.1%)	89 (4.7%)	222 (11.4%)	750 (39.8%)
BP-lowering medication, n (%)	96 (4.3%)	305 (13.2%)	664 (29.5%)	158 (13.2%)	299 (24.1%)	449 (37.4%)	596 (31.6%)	895 (46.1%)	1,129 (59.9%)
Lipid-lowering medication, n (%)	50 (2.2%)	73 (3.1%)	126 (5.6%)	124 (10.3%)	208 (16.8%)	305 (25.4%)	509 (27.0%)	672 (34.6%)	761 (40.4%)
Glucose-lowering medication, n (%)	14 (0.6%)	32 (1.4%)	147 (6.5%)	6 (0.5%)	29 (2.3%)	150 (12.5%)	67 (3.6%)	162 (8.4%)	531 (28.2%)
Metabolic biomarkers, mean (SD)									
SBP (mmHg)	110.8 (9.7)	127.0 (10.1)	149.7 (18.7)	116.1 (8.9)	131.4 (8.0)	148.1 (14.1)	112.7 (11.0)	130.0 (10.5)	147.6 (17.9)
HbA1c (%)	4.9 (0.4)	5.1 (0.4)	5.7 (1.1)	5.5 (0.3)	5.7 (0.3)	6.2 (0.9)	5.3 (0.4)	5.6 (0.4)	6.4 (1.3)
non-HDL-C (mmol/L)	3.4 (0.9)	3.7 (1.0)	3.9 (1.0)	4.1 (1.1)	4.2 (1.1)	4.1 (1.2)	3.9 (0.9)	4.0 (0.9)	4.0 (1.0)
BMI (kg/m^2^)	22.9 (14.4)	24.7 (51.8)	24.7 (9.5)	26.5 (4.4)	28.1 (4.7)	29.6 (5.3)	28.1 (5.4)	29.4 (5.4)	30.7 (5.9)

BMI, body mass index; BP, blood pressure; CHARLS, China Health and Retirement Longitudinal Study; ELSA, English Longitudinal Study of Ageing; HbA1c, hemoglobin A1c; HRS, Health and Retirement Study; HSI, hemoglobin A1c-systolic blood pressure index; non-HDL-C, non-high density lipoprotein cholesterol; SBP, systolic blood pressure.

### Associations between baseline and cumulative HSI levels and incident CVD

**Figure 2:**
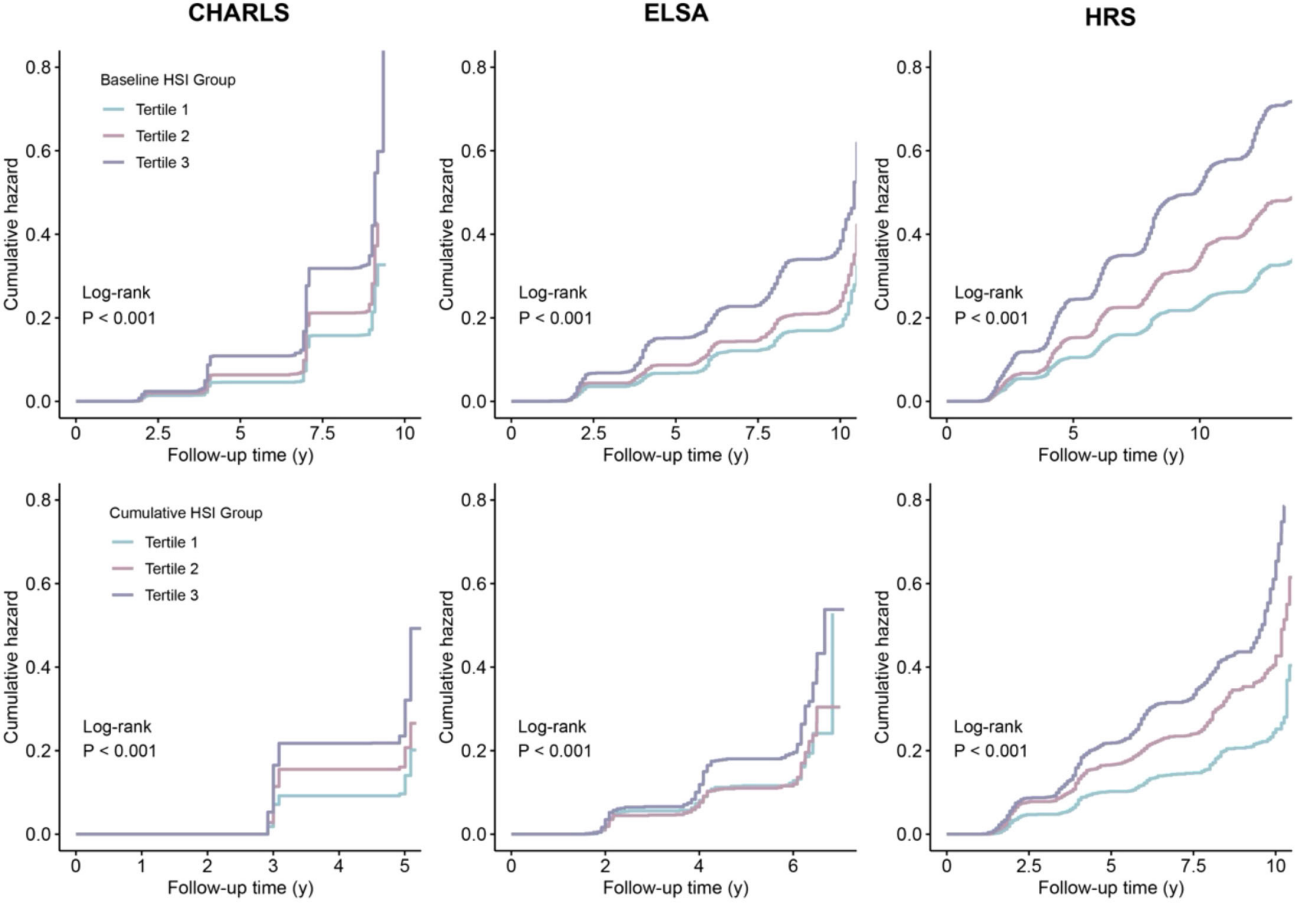
Cumulative hazard curve plots of incident cardiovascular disease according to the baseline and cumulative HSI tertiles. CHARLS, China Health and Retirement Longitudinal Study; ELSA, English Longitudinal Study of Ageing; HR, hazard ratio; HRS, Health and Retirement Study.

**Figure 3:**
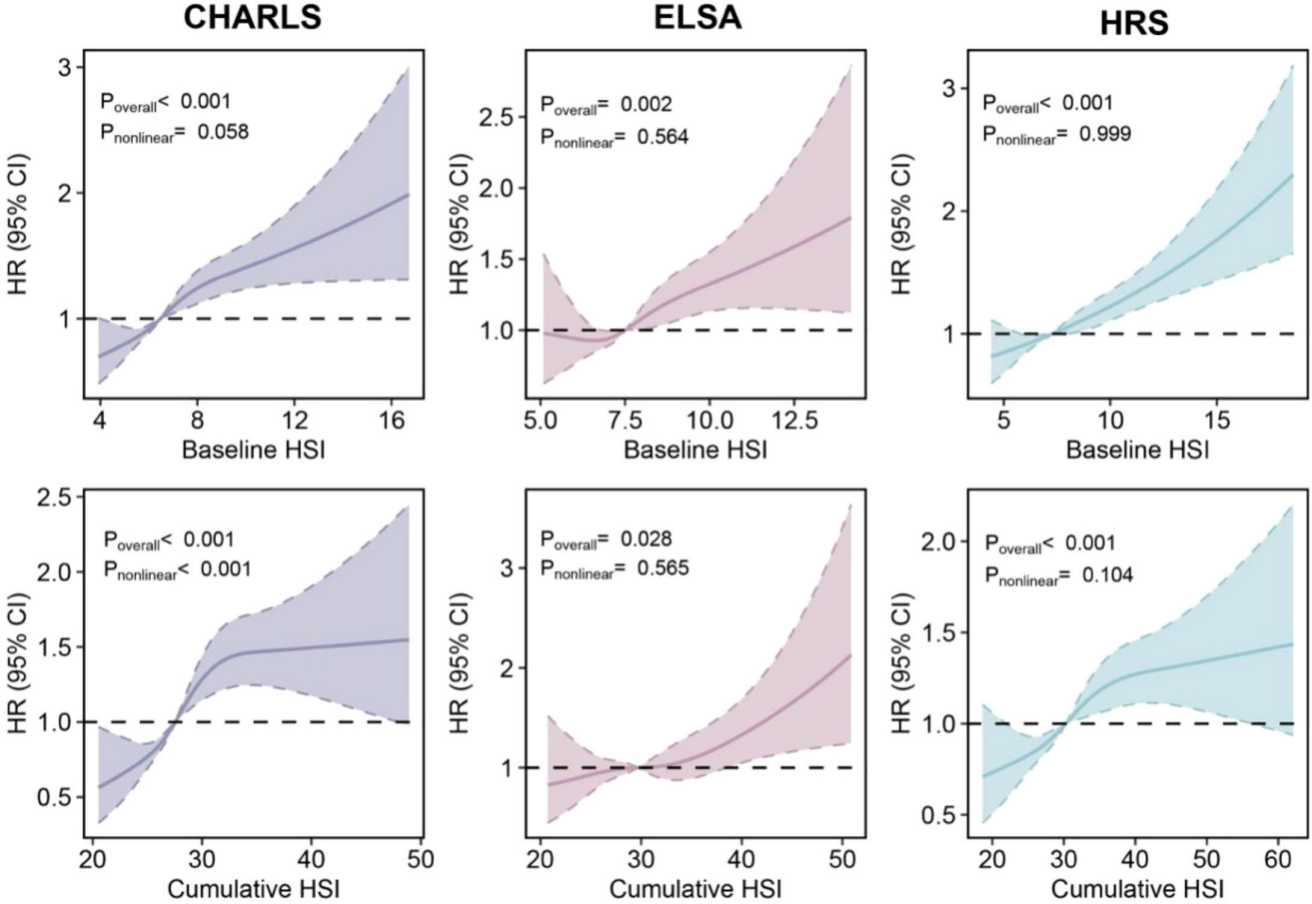
Dose-response associations between baseline and cumulative HSI levels and incident cardiovascular disease. Restricted cubic spline regression, adjusted for age, sex, and ethnicity, education, socioeconomic status, smoking status, alcohol consumption, non-high density lipoprotein cholesterol, body mass index, and current medications for hypertension, cholesterol, and diabetes, was used to assess the dose-response associations. The dashed lines indicate the null line. CHARLS, China Health and Retirement Longitudinal Study; CI, confidence interval; ELSA, English Longitudinal Study of Ageing; HR, hazard ratio; HRS, Health and Retirement Study; HSI, hemoglobin A1c-systolic blood pressure index.

Elevated HbA1c and SBP levels were independently associated with increased CVD risk across all three cohorts, with their combination conferring greater risk than either component alone (**Supplementary Table S2**). Higher baseline HSI levels were significantly associated with increased CVD risk in CHARLS (HR per 1 SD increase = 1.16, 95% CI 1.11–1.22), ELSA (HR = 1.13, 95% CI 1.06–1.21), and HRS (HR = 1.14, 95% CI 1.10–1.19). Also, elevated cumulative HSI levels were associated with increased CVD risk (CHARLS: HR per 1 SD increase = 1.19, 95% CI 1.12–1.26; ELSA: 1.14, 95% CI 1.04–1.26; HRS: 1.15, 95% CI 1.08–1.22) (**Table 2**). These associations remained consistent across models with different covariate adjustments. No evidence of nonlinear associations was observed between baseline HSI and incident CVD (*P*_nonlinear_ for CHARLS = 0.058; *P*_nonlinear_ for ELSA = 0.564; *P*_nonlinear_ for HRS = 0.999), although the association between cumulative HSI and incident CVD in CHARLS showed nonlinearity (*P*_nonlinear_ < 0.001). The dose-response relationships between both baseline and cumulative HSI and incident CVD demonstrated consistent monotonic trends (**Figure 2**). Cumulative hazard curves showed that participants in the highest HSI tertile had a significantly higher incidence of CVD than those in the lowest tertile across all three cohorts (all *P* < 0.001) (**Figure 3**). The optimal cutoff value of HSI was 8.0, and the cumulative hazard curves confirmed the discriminatory value of HSI cutoff value for cardiovascular risk stratification (**Supplementary Figure S2**).

**Table 2:** Associations between baseline and cumulative HSI levels and incident cardiovascular disease.

Cohorts	HR (95% CI)			*P* for trend	HR per 1 SD increase (95% CI)	*P* value
	Tertile 1	Tertile 2	Tertile 3			
Baseline HSI						
CHARLS						
Model 0	1.00 [Reference]	1.33 (1.18–1.51)	2.00 (1.78–2.25)	<0.001	1.28 (1.23–1.32)	<0.001
Model 1	1.00 [Reference]	1.28 (1.13–1.45)	1.78 (1.58–2.01)	<0.001	1.23 (1.19–1.28)	<0.001
Model 2	1.00 [Reference]	1.21 (1.07–1.38)	1.50 (1.32–1.70)	<0.001	1.16 (1.11–1.22)	<0.001
ELSA						
Model 0	1.00 [Reference]	1.30 (1.09–1.54)	2.00 (1.71–2.35)	<0.001	1.30 (1.23–1.37)	<0.001
Model 1	1.00 [Reference]	1.08 (0.91–1.29)	1.45 (1.23–1.71)	<0.001	1.18 (1.11–1.25)	<0.001
Model 2	1.00 [Reference]	1.03 (0.86–1.23)	1.29 (1.08–1.53)	0.002	1.13 (1.06–1.21)	<0.001
HRS						
Model 0	1.00 [Reference]	1.47 (1.32–1.64)	2.17 (1.95–2.41)	<0.001	1.27 (1.23–1.31)	<0.001
Model 1	1.00 [Reference]	1.20 (1.08–1.34)	1.50 (1.35–1.67)	<0.001	1.20 (1.16–1.25)	<0.001
Model 2	1.00 [Reference]	1.14 (1.02–1.28)	1.29 (1.15–1.44)	<0.001	1.14 (1.10–1.19)	<0.001
Cumulative HSI						
CHARLS						
Model 0	1.00 [Reference]	1.45 (1.21–1.73)	2.34 (1.98–2.76)	<0.001	1.32 (1.25-1.38)	<0.001
Model 1	1.00 [Reference]	1.38 (1.15–1.65)	2.10 (1.77–2.49)	<0.001	1.28 (1.22-1.35)	<0.001
Model 2	1.00 [Reference]	1.28 (1.07–1.54)	1.75 (1.46–2.09)	<0.001	1.19 (1.12-1.26)	<0.001
ELSA						
Model 0	1.00 [Reference]	1.04 (0.81–1.32)	1.61 (1.28–2.01)	<0.001	1.28 (1.18–1.38)	<0.001
Model 1	1.00 [Reference]	0.90 (0.70–1.15)	1.29 (1.03–1.63)	0.016	1.20 (1.10–1.30)	<0.001
Model 2	1.00 [Reference]	0.84 (0.66–1.08)	1.13 (0.88–1.44)	0.234	1.14 (1.04–1.26)	0.006
HRS						
Model 0	1.00 [Reference]	1.67 (1.43–1.96)	2.28 (1.96–2.66)	<0.001	1.27 (1.22–1.34)	<0.001
Model 1	1.00 [Reference]	1.36 (1.15–1.59)	1.72 (1.47–2.02)	<0.001	1.21 (1.14–1.27)	<0.001
Model 2	1.00 [Reference]	1.27 (1.08–1.50)	1.49 (1.26–1.76)	<0.001	1.15 (1.08–1.22)	<0.001

Cox proportional hazards regression was used to assess the associations between HSI and incident cardiovascular disease. Model 0 was unadjusted for any covariates, Model 1 was adjusted for basic demographic characteristics including age, sex, and ethnicity, while Model 2 incorporated additional socioeconomic and clinical variables beyond the demographic factors, including education level, socioeconomic status, smoking status, alcohol consumption, non-high-density lipoprotein cholesterol, body mass index, and current use of medications for hypertension, cholesterol management, and diabetes. CHARLS, China Health and Retirement Longitudinal Study; CI, confidence interval; ELSA, English Longitudinal Study of Ageing; HR, hazard ratio; HRS, Health and Retirement Study; HSI, hemoglobin A1c-systolic blood pressure index.

### Comparison of predictive values of HSI and its components for incident CVD

**Figure 4:**
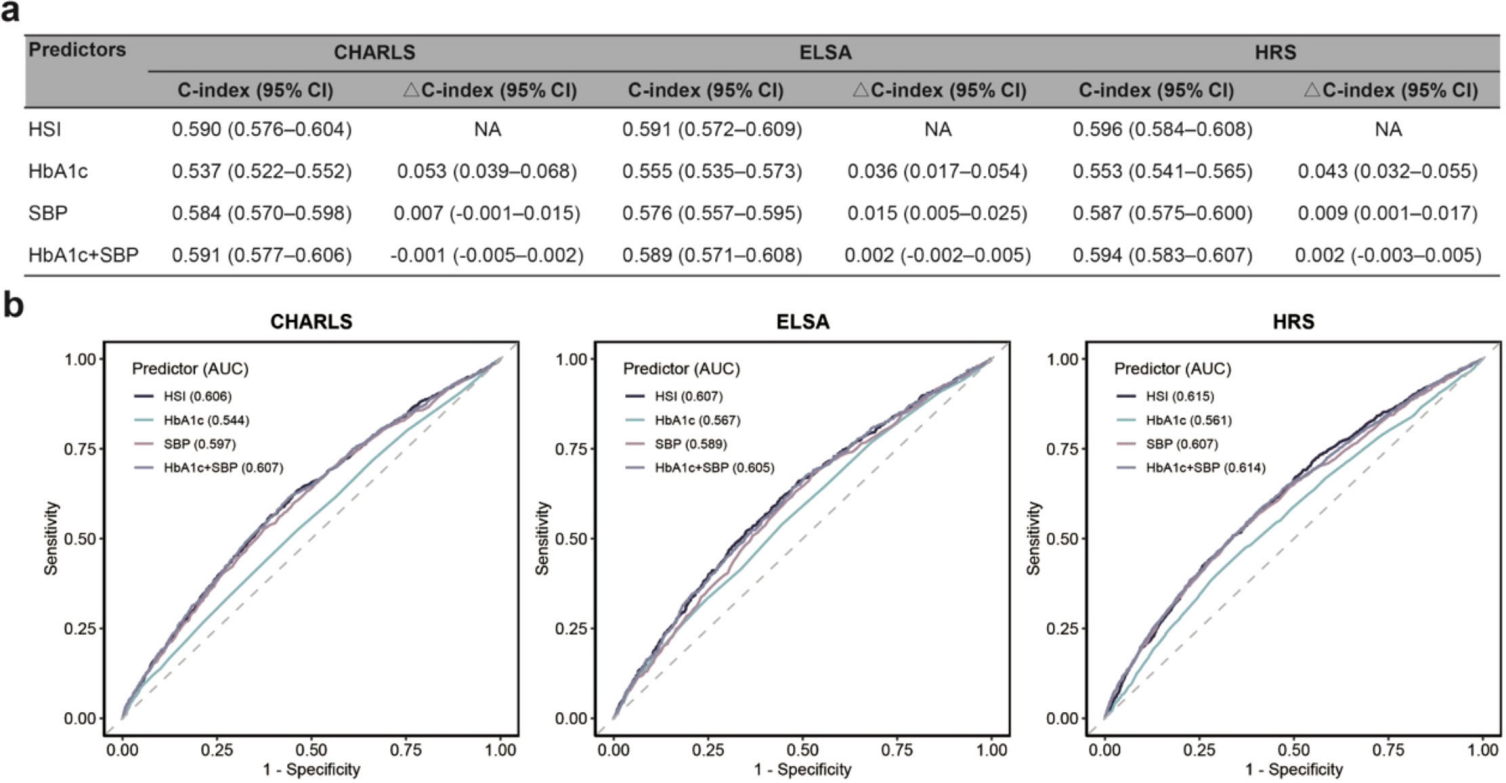
Predictive performance of HSI for incident cardiovascular disease. **(a)** Harrell’s concordance index (C-index) of systolic blood pressure (SBP), hemoglobin A1c (HbA1c), their additive combination, and HbA1c-SBP index (HSI) was calculated and its 95% confidence interval (CI) was estimated using bootstrap resampling with 1,000 iterations. Their additive combination indicates the model including both SBP and HbA1c. **(b)** 10-year receiver operating characteristic curves were depicted. Due to the max follow-up time of China Health and Retirement Longitudinal Study (CHARLS) less than 10 years, 9-year receiver operating characteristic curve was presented. AUC, area under the curve; ELSA, English Longitudinal Study of Ageing; HRS, Health and Retirement Study.

HSI demonstrated moderate predictive performance for incident CVD in CHARLS (C-index = 0.590, 95% CI 0.576–0.604), ELSA (C-index = 0.591, 95% CI 0.572–0.609), and HRS (C-index = 0.596, 95% CI 0.584–0.608). HSI significantly enhanced the predictive value compared with HbA1c alone (CHARLS: ΔC-index = 0.053, 95% CI 0.039–0.068; ELSA: 0.036, 95% CI 0.017–0.054; HRS: 0.043, 95% CI 0.032–0.055) and modestly improved predictive performance compared with SBP alone (CHARLS: ΔC-index = 0.007, 95% CI -0.001–0.015; ELSA: 0.015, 95% CI 0.005–0.025; HRS: 0.009, 95% CI 0.001–0.017) (**Figure 4a**). The predictive performance of HSI was comparable to that of models including both HbA1c and SBP simultaneously (CHARLS: ΔC-index = -0.001, 95% CI -0.005–0.002; ELSA: 0.002, 95% CI -0.002–0.005; HRS: 0.002, 95% CI -0.003–0.005). These findings were consistent across ROC curve analyses in all three cohorts (**Figure 4b**).

### Sensitivity analyses

Subgroup analyses demonstrated that associations between both baseline and cumulative HSI and incident CVD were almost consistent across diverse populations (**Supplementary Tables S3**, **S4**). Age attenuated the association between baseline HSI and incident CVD in CHARLS (P for interaction = 0.002). However, both the associations among individuals aged <60 years and those aged ≥60 years were significant (<60 years: HR = 1.19, 95% CI: 1.12–1.26; ≥60 years: HR = 1.14, 95% CI: 1.07–1.22). The diabetes status diversely modified the associations between cumulative HSI and incident CVD across three cohorts (CHARLS: *P* for interaction <0.001; ELSA: *P* for interaction =0.027; HRS: *P* for interaction =0.011). Furthermore, the results remained unchanged when participants with events occurring within the first 2 years of follow-up were excluded, or when Fine-Gray competing risk regression or modified Poisson regression were applied (**Supplementary Tables S5–S7**). These sensitivity analyses further validated the robustness of HSI. Cumulative hazard curves based on clinical thresholds demonstrated that clinical HSI cutpoints effectively stratified cardiovascular risk (all *P* < 0.001) (**Supplementary Figure S3**).

## Discussion

This study used a simple calculation method to construct HSI, a novel composite biomarker integrating glycemic control and blood pressure status for cardiovascular risk assessment. Our results demonstrated that baseline and cumulative HSI were consistently associated with incident CVD across three cohorts of China, UK, and US, and maintained robust associations across diverse demographic and clinical subgroups. HSI exhibited moderate performance in predicting cardiovascular risk and was superior to its individual components. This study supported the generalizability of HSI as a cardiovascular risk assessment tool across diverse populations.

Previous observational studies have shown that concurrent diabetes and hypertension jointly contributed to the accelerated arterial stiffening and increased CVD risks (6, 7, 18–21). Mendelian randomization studies revealed that SBP and HbA1c were two established biomarkers causally associated with CVD risk (22–25). Numerous randomized controlled trials have shown that pharmacological lowering of SBP and HbA1c significantly decreased the risk of cardiovascular events (12, 26–29). The integrated management of hypertension and diabetes for decreasing cardiovascular risk was increasingly emphasized because of internal complex interplay among metabolic factors (3–5).

Diabetes and hypertension share common pathophysiological pathways, including overactivation of the renin-angiotensin-aldosterone system, increased oxidative stress, chronic inflammation, reduced insulin-mediated blood vessel relaxation, heightened sympathetic nervous system activity, disrupted immune responses, and impaired kidney sodium handling (30–32). These evidence constitutes the biological basis for combining HbA1c and SBP in a composite index. The present study revealed that HSI, a composite indicator of HbA1c and SBP, was consistently associated with increased CVD risks across various population, confirming the importance of comprehensive glycemic and blood pressure control. Furthermore, cumulative HSI burden was also shown to be associated with higher CVD risks, highlighting the need for early and sustained management.

Numerous observational studies have investigated the predictive value of fasting blood glucose-related indices for incident CVD (33–35). Although fasting blood glucose is easy to obtain and cost-effective, it is susceptible to food and medication effects, with high biological variation. In contrast, HbA1c is more stable and reflects long-term blood glucose control. A Mendelian randomization study demonstrated that HbA1c rather than fasting blood glucose was causally associated with an increased risk of coronary heart disease (24). The clinical guidelines recommend both HbA1c and fasting blood glucose for diagnosing diabetes; however, only HbA1c is used for risk prediction (10, 36). This preference is also reflected in major cardiovascular risk assessment tools: both the European Society of Cardiology’s SCORE2-Diabetes equations and the American Heart Association’s PREVENT equations incorporate HbA1c as a predictor for incident CVD (37, 38).

The development of HSI has filled a gap in current clinical practice by providing a single metric that simultaneously captures glycemic control and blood pressure status. This study established HSI as a valuable clinical tool for cardiovascular risk assessment, demonstrating consistent predictive performance across diverse populations (China, the UK, and the US) with superior accuracy compared with individual components. The identified optimal cut-off value of 8.0 provides clinicians with a practical threshold for risk stratification, whereas the robust associations across different demographic subgroups ensure broad clinical applicability. HSI offers a simple, reliable approach for identifying high-risk patients and guiding personalized cardiovascular prevention strategies.

This study demonstrated solid methodological design through its large sample size, extended follow-up periods, and multi-population validation across three continents. The comprehensive analysis of both baseline and cumulative HSI values, along with thorough sensitivity analyses using various statistical approaches, provides reliable evidence for the predictive validity of HSI. The consistent associations observed across diverse demographic and clinical subgroups, combined with multiple validation methods, support the applicability and reliability of HSI as a cardiovascular risk assessment tool in clinical practice.

In the era of artificial intelligence (AI)-enabled cardiovascular prevention, HSI has strong potential for integration into broader clinical decision-support frameworks. Because both HbA1c and SBP are routinely measured in clinical practice, HSI can be automatically calculated within electronic health record systems and incorporated as a scalable and interpretable input for AI-driven risk stratification models. Furthermore, cumulative HSI assessment enables longitudinal risk monitoring, facilitating dynamic evaluation of cardiometabolic burden and early identification of individuals who may benefit from intensified preventive strategies. Importantly, reliable identification of high-risk individuals using HSI also creates opportunities to guide targeted pharmacological and lifestyle interventions (39, 40).

This study had several limitations. First, the cardiovascular outcomes were primarily based on self-reported data, which might have introduced reporting bias and potential misclassification of events, potentially affecting the accuracy of outcome assessment. Second, being an observational study, it could not establish causal relationships between HSI and CVD risk, only demonstrating statistical associations influenced by unmeasured confounding factors. Third, the study populations were derived from epidemiological cohorts rather than clinical settings, which might have limited the direct applicability of findings to clinical practice. Prospective clinical trials and validation studies in real-world clinical environments are needed to confirm the clinical utility of HSI and establish its role in routine cardiovascular risk management protocols.

## Conclusions

HSI represented a novel, clinically relevant composite biomarker effectively integrating glycemic control and blood pressure status for cardiovascular risk assessment. Our findings demonstrated consistent associations between HSI and incident CVD across diverse populations, with superior predictive performance compared with individual components. The simplicity and clinical relevance of HSI make it a potentially valuable tool for cardiovascular risk stratification in clinical practice.

## Data Availability

The original contributions presented in the study are included in the article/**Supplementary Material**. Further inquiries can be directed to the corresponding authors.

## References

[1] Mensah GA Fuster V Murray CJL Roth GA . Global burden of cardiovascular diseases and risks, 1990-2022. J Am Coll Cardiol. (2023) 82:2350–473. doi: 10.1016/j.jacc.2023.11.007, PMID: 38092509 PMC7615984

[2] Vaduganathan M Mensah GA Turco JV Fuster V Roth GA . The global burden of cardiovascular diseases and risk: A compass for future health. J Am Coll Cardiol. (2022) 80:2361–71. doi: 10.1016/j.jacc.2022.11.005, PMID: 36368511

[3] Lloyd-Jones DM Allen NB Anderson CAM Black T Brewer LC Foraker RE . Life’s essential 8: updating and enhancing the american heart association’s construct of cardiovascular health: A presidential advisory from the american heart association. Circulation. (2022) 146:e18–43. doi: 10.1161/cir.0000000000001078, PMID: 35766027 PMC10503546

[4] Ndumele CE Rangaswami J Chow SL Neeland IJ Tuttle KR Khan SS . Cardiovascular-kidney-metabolic health: A presidential advisory from the american heart association. Circulation. (2023) 148:1606–35. doi: 10.1161/cir.0000000000001184, PMID: 37807924

[5] Ndumele CE Neeland IJ Tuttle KR Chow SL Mathew RO Khan SS . A synopsis of the evidence for the science and clinical management of cardiovascular-kidney-metabolic (CKM) syndrome: A scientific statement from the american heart association. Circulation. (2023) 148:1636–64. doi: 10.1161/cir.0000000000001186, PMID: 37807920

[6] Yuan Y Isasi CR Al-Rousan T Ghosh AK Mullachery PH Palta P . Associations of concurrent hypertension and type 2 diabetes with mortality outcomes: A prospective study of U.S. Adults. Diabetes Care. (2025) 48:1241–50. doi: 10.2337/dca24-0118, PMID: 40397766 PMC12178617

[7] Suzuki Y Kaneko H Yano Y Okada A Itoh H Matsuoka S . Interaction of blood pressure and glycemic status in developing cardiovascular disease: analysis of a nationwide real-world database. J Am Heart Assoc. (2023) 12:e026192. doi: 10.1161/jaha.122.026192, PMID: 36565182 PMC9973580

[8] McEvoy JW McCarthy CP Bruno RM Brouwers S Canavan MD Ceconi C . 2024 ESC Guidelines for the management of elevated blood pressure and hypertension. Eur Heart J. (2024) 45:3912–4018. doi: 10.1093/eurheartj/ehae178, PMID: 39210715

[9] Jones DW Ferdinand KC Taler SJ Johnson HM Shimbo D Abdalla M . 2025 AHA/ACC/AANP/AAPA/ABC/ACCP/ACPM/AGS/AMA/ASPC/NMA/PCNA/SGIM guideline for the prevention, detection, evaluation and management of high blood pressure in adults: A report of the american college of cardiology/american heart association joint committee on clinical practice guidelines. Circulation. (2025) 152:e114–e218. doi: 10.1161/cir.0000000000001356, PMID: 40811497

[10] 2. Diagnosis and classification of diabetes: standards of care in diabetes-2024. Diabetes Care. (2024) 47:S20–s42. doi: 10.2337/dc24-S002, PMID: 38078589 PMC10725812

[11] Davies MJ Aroda VR Collins BS Gabbay RA Green J Maruthur NM . Management of hyperglycaemia in type 2 diabetes, 2022. A consensus report by the American Diabetes Association (ADA) and the European Association for the Study of Diabetes (EASD). Diabetologia. (2022) 65:1925–66. doi: 10.1007/s00125-022-05787-2, PMID: 36151309 PMC9510507

[12] Pharmacological blood pressure lowering for primary and secondary prevention of cardiovascular disease across different levels of blood pressure: an individual participant-level data meta-analysis. Lancet. (2021) 397:1625–36. doi: 10.1016/s0140-6736(21)00590-0, PMID: 33933205 PMC8102467

[13] Welsh C Welsh P Celis-Morales CA Mark PB Mackay D Ghouri N . Glycated hemoglobin, prediabetes, and the links to cardiovascular disease: data from UK biobank. Diabetes Care. (2020) 43:440–5. doi: 10.2337/dc19-1683, PMID: 31852727

[14] Wan EYF Fung CSC Wong CKH Chin WY Lam CLK . Association of hemoglobin A1c levels with cardiovascular disease and mortality in chinese patients with diabetes. J Am Coll Cardiol. (2016) 67:456–8. doi: 10.1016/j.jacc.2015.11.020, PMID: 26821636

[15] Pencina KM Thanassoulis G Pencina MJ Toth PP Sniderman AD . Hemoglobin A1c and abdominal obesity as predictors of diabetes and ASCVD in individuals with prediabetes in UK Biobank: a prospective observational study. Cardiovasc Diabetol. (2024) 23:448. doi: 10.1186/s12933-024-02525-3, PMID: 39702291 PMC11660550

[16] Hoelzel W Weykamp C Jeppsson JO Miedema K Barr JR Goodall I . IFCC reference system for measurement of hemoglobin A1c in human blood and the national standardization schemes in the United States, Japan, and Sweden: a method-comparison study. Clin Chem. (2004) 50:166–74. doi: 10.1373/clinchem.2003.024802, PMID: 14709644

[17] van Buuren S Groothuis-Oudshoorn K . mice: multivariate imputation by chained equations in R. J Stat Softw. (2011) 45:1–67. doi: 10.18637/jss.v045.i03

[18] Wang J Wang Z Guo F Zhang Y Ji H Chen G . Individual and combined cardiometabolic morbidities and the subsequent risk of cardiovascular events in chinese adults. J Clin Endocrinol Metab. (2022) 107:e84–94. doi: 10.1210/clinem/dgab609, PMID: 34427675

[19] Imai Y Hirata T Saitoh S Ninomiya T Miyamoto Y Ohnishi H . Impact of hypertension stratified by diabetes on the lifetime risk of cardiovascular disease mortality in Japan: a pooled analysis of data from the Evidence for Cardiovascular Prevention from Observational Cohorts in Japan study. Hypertens Res. (2020) 43:1437–44. doi: 10.1038/s41440-020-0502-5, PMID: 32620896

[20] Yen FS Wei JC Chiu LT Hsu CC Hwu CM . Diabetes, hypertension, and cardiovascular disease development. J Transl Med. (2022) 20:9. doi: 10.1186/s12967-021-03217-2, PMID: 34980154 PMC8722333

[21] Tomiyama H Hashimoto H Hirayama Y Yambe M Yamada J Koji Y . Synergistic acceleration of arterial stiffening in the presence of raised blood pressure and raised plasma glucose. Hypertension. (2006) 47:180–8. doi: 10.1161/01.HYP.0000198539.34501.1a, PMID: 16380535

[22] Pozarickij A Gan W Lin K Clarke R Fairhurst-Hunter Z Koido M . Causal relevance of different blood pressure traits on risk of cardiovascular diseases: GWAS and Mendelian randomisation in 100,000 Chinese adults. Nat Commun. (2024) 15:6265. doi: 10.1038/s41467-024-50297-x, PMID: 39048560 PMC11269703

[23] Ma Y Wang M Chen X Yao J Ding Y Gao Q . Effect of the blood pressure and antihypertensive drugs on cerebral small vessel disease: A mendelian randomization study. Stroke. (2024) 55:1838–46. doi: 10.1161/strokeaha.123.045664, PMID: 38818733

[24] Ross S Gerstein HC Eikelboom J Anand SS Yusuf S Paré G . Mendelian randomization analysis supports the causal role of dysglycaemia and diabetes in the risk of coronary artery disease. Eur Heart J. (2015) 36:1454–62. doi: 10.1093/eurheartj/ehv083, PMID: 25825043

[25] Leong A Chen J Wheeler E Hivert MF Liu CT Merino J . Mendelian randomization analysis of hemoglobin A(1c) as a risk factor for coronary artery disease. Diabetes Care. (2019) 42:1202–8. doi: 10.2337/dc18-1712, PMID: 30659074 PMC6609962

[26] Brunström M Carlberg B . Association of blood pressure lowering with mortality and cardiovascular disease across blood pressure levels: A systematic review and meta-analysis. JAMA Intern Med. (2018) 178:28–36. doi: 10.1001/jamainternmed.2017.6015, PMID: 29131895 PMC5833509

[27] Marso SP Daniels GH Brown-Frandsen K Kristensen P Mann JF Nauck MA . Liraglutide and cardiovascular outcomes in type 2 diabetes. N Engl J Med. (2016) 375:311–22. doi: 10.1056/NEJMoa1603827, PMID: 27295427 PMC4985288

[28] Zinman B Wanner C Lachin JM Fitchett D Bluhmki E Hantel S . Empagliflozin, cardiovascular outcomes, and mortality in type 2 diabetes. N Engl J Med. (2015) 373:2117–28. doi: 10.1056/NEJMoa1504720, PMID: 26378978

[29] Turnbull FM Abraira C Anderson RJ Byington RP Chalmers JP Duckworth WC . Intensive glucose control and macrovascular outcomes in type 2 diabetes. Diabetologia. (2009) 52:2288–98. doi: 10.1007/s00125-009-1470-0, PMID: 19655124

[30] Lastra G Syed S Kurukulasuriya LR Manrique C Sowers JR . Type 2 diabetes mellitus and hypertension: an update. Endocrinol Metab Clin North Am. (2014) 43:103–22. doi: 10.1016/j.ecl.2013.09.005, PMID: 24582094 PMC3942662

[31] Hezam AAM Shaghdar HBM Chen L . The connection between hypertension and diabetes and their role in heart and kidney disease development. J Res Med Sci. (2024) 29:22. doi: 10.4103/jrms.jrms_470_23, PMID: 38855561 PMC11162087

[32] Lleva RR Inzucchi SE . Glucose, blood pressure, and cardiovascular risk. Circ Cardiovasc Qual Outcom. (2012) 5:145–7. doi: 10.1161/circoutcomes.112.965046, PMID: 22438461

[33] Li W Shen C Kong W Zhou X Fan H Zhang Y . Association between the triglyceride glucose-body mass index and future cardiovascular disease risk in a population with Cardiovascular-Kidney-Metabolic syndrome stage 0-3: a nationwide prospective cohort study. Cardiovasc Diabetol. (2024) 23:292. doi: 10.1186/s12933-024-02352-6, PMID: 39113004 PMC11308445

[34] Dang K Wang X Hu J Zhang Y Cheng L Qi X . The association between triglyceride-glucose index and its combination with obesity indicators and cardiovascular disease: NHANES 2003-2018. Cardiovasc Diabetol. (2024) 23:8. doi: 10.1186/s12933-023-02115-9, PMID: 38184598 PMC10771672

[35] Zhou H Ding X Lan Y Chen S Wu S Wu D . Multi-trajectories of triglyceride-glucose index and lifestyle with Cardiovascular Disease: a cohort study. Cardiovasc Diabetol. (2023) 22:341. doi: 10.1186/s12933-023-02076-z, PMID: 38093279 PMC10720233

[36] Committee ADAPP . 6. Glycemic goals and hypoglycemia: standards of care in diabetes—2025. Diabetes Care. (2024) 48:S128–45. doi: 10.2337/dc25-S006, PMID: 39651981 PMC11635034

[37] Khan SS Matsushita K Sang Y Ballew SH Grams ME Surapaneni A . Development and validation of the american heart association’s PREVENT equations. Circulation. (2024) 149:430–49. doi: 10.1161/circulationaha.123.067626, PMID: 37947085 PMC10910659

[38] SCORE2-Diabetes: 10-year cardiovascular risk estimation in type 2 diabetes in Europe. Eur Heart J. (2023) 44:2544–56. doi: 10.1093/eurheartj/ehad260, PMID: 37247330 PMC10361012

[39] Lucke-Wold B Zasler ND Ruchika F Weisman S Le D Brunicardi J . Supplement and nutraceutical therapy in traumatic brain injury. Nutr Neurosci. (2025) 28:709–43. doi: 10.1080/1028415x.2024.2404782, PMID: 40440029

[40] Segherlou ZH Shakeri-Darzekonani M Khavandegar A Stephenson S Ciccone K Masheghati F . Hormonal influences on cerebral aneurysms: unraveling the complex connections. Expert Rev Endocrinol Metab. (2024) 19:207–15. doi: 10.1080/17446651.2024.2347275, PMID: 38712738

