# Supplementary material for: Hemoglobin A1c-systolic blood pressure index as a novel predictor of cardiovascular disease: evidence from three prospective cohorts
Source: Front Endocrinol (Lausanne). 2026 Feb 18;17:1718936. doi: 10.3389/fendo.2026.1718936 (PMC12956636; doi:10.3389/fendo.2026.1718936)
Supplement: Supplementary file 1 [file DataSheet1.docx]

**Supplementary Materials**

[Supplementary Methods 2](#_Toc208105124)

[Table S1. Baseline characteristics of participants for cumulative HSI analyses. 5](#_Toc208105125)

[Table S2. Associations between the individual and combination of HbA1c and SBP and incident cardiovascular disease. 6](#_Toc208105126)

[Table S3. Associations between baseline HSI levels and incident cardiovascular disease across diverse subgroups. 7](#_Toc208105127)

[Table S4. Associations between cumulative HSI levels and incident cardiovascular disease across diverse subgroups. 9](#_Toc208105128)

[Table S5. Associations between baseline and cumulative HSI levels and incident cardiovascular disease after excluding participants who developed CVD within the first two years of follow-up. 11](#_Toc208105129)

[Table S6. Associations between baseline and cumulative HSI levels and incident cardiovascular disease using Fine-Gray regression. 12](#_Toc208105130)

[Table S7. Associations between baseline and cumulative HSI levels and incident cardiovascular disease using modified Poisson regression. 13](#_Toc208105131)

[Figure S1. Timelines of study design. 14](#_Toc208105132)

[Figure S2. Cumulative hazard curve plots of incident cardiovascular disease according to the optimal cutpoint of baseline HSI. 15](#_Toc208105133)

[Figure S3. Cumulative hazard curve plots of incident cardiovascular disease according to the clinical cutpoints of baseline HSI. 16](#_Toc208105134)

## **Supplementary Methods**

**1. China Health and Retirement Longitudinal Study (CHARLS):**

CHARLS is a nationally representative longitudinal survey of Chinese residents aged 45 years and older, initiated in 2011 by Peking University. It collects detailed information on demographics, health status, physical functioning, cognition, health behaviors, and economic circumstances. The study aims to provide high-quality data to support research on aging, health, and socioeconomic factors in China. Data are collected through structured interviews, physical measurements, and biomarker assessments, and follow-up waves are conducted every two to three years.

**2. English Longitudinal Study of Ageing (ELSA):**

ELSA is a prospective cohort study of individuals aged 50 and older living in England, launched in 2002. It is designed to investigate the health, social, and economic circumstances of older adults. The study collects a wide range of information, including physical health, cognitive function, psychosocial factors, biomarkers, and financial well-being, through regular interviews and health assessments. ELSA data provide valuable insights into aging processes, health inequalities, and policy-relevant research in the UK.

**3. Health and Retirement Study (HRS):**

HRS is a large-scale longitudinal study of Americans over the age of 50, started in 1992 and sponsored by the U.S. National Institute on Aging. The study collects comprehensive information on health, retirement, income, wealth, family structure, and psychosocial factors. HRS includes regular biennial interviews, physical and cognitive assessments, and biomarker data collection. Its rich dataset enables researchers to study the interplay between aging, health, and economic outcomes in the United States.

## **Table S1. Baseline characteristics of participants for cumulative HSI analyses.**

| **Characteristics** | **Cumulative HSI (CHARLS)** | | | **Cumulative HSI (ELSA)** | | | **Cumulative HSI (HRS)** | | |
| --- | --- | --- | --- | --- | --- | --- | --- | --- | --- |
|  | Tertile 1 | Tertile 2 | Tertile 3 | Tertile 1 | Tertile 2 | Tertile 3 | Tertile 1 | Tertile 2 | Tertile 3 |
| Number | 1459 | 1503 | 1459 | 849 | 873 | 849 | 1268 | 1306 | 1268 |
| Age (years), mean (SD) | 54.8 (8.2) | 57.4 (8.5) | 59.6 (8.7) | 60.8 (6.7) | 63.0 (7.8) | 64.9 (7.4) | 61.3 (8.1) | 64.5 (8.4) | 66.2 (8.4) |
| Men, n (%) | 621 (42.6%) | 694 (46.2%) | 659 (45.2%) | 354 (41.7%) | 405 (46.4%) | 393 (46.3%) | 343 (27.1%) | 513 (39.3%) | 527 (41.6%) |
| White, n (%) | 0 (0%) | 0 (0%) | 0 (0%) | 838 (98.7%) | 849 (97.3%) | 830 (97.8%) | 1142 (90.1%) | 1096 (83.9%) | 955 (75.3%) |
| High school education, n (%) | 154 (10.6%) | 137 (9.1%) | 98 (6.7%) | 671 (79.0%) | 665 (76.2%) | 621 (73.1%) | 1175 (92.7%) | 1113 (85.2%) | 1006 (79.3%) |
| Socioeconomic deprivation, n (%) | 158 (10.8%) | 188 (12.5%) | 210 (14.4%) | 120 (14.1%) | 131 (15.0%) | 142 (16.7%) | 682 (53.8%) | 682 (52.2%) | 678 (53.5%) |
| Smoking, n (%) | 434 (29.7%) | 449 (29.9%) | 439 (30.1%) | 95 (11.2%) | 97 (11.1%) | 112 (13.2%) | 133 (10.5%) | 163 (12.5%) | 146 (11.5%) |
| Alcohol consumption, n (%) | 418 (28.6%) | 498 (33.1%) | 423 (29.0%) | 794 (93.5%) | 814 (93.2%) | 786 (92.6%) | 558 (44.0%) | 536 (41.0%) | 428 (33.8%) |
| Hypertension, n (%) | 155 (10.6%) | 474 (31.5%) | 925 (63.4%) | 166 (19.6%) | 419 (48.0%) | 657 (77.4%) | 430 (33.9%) | 821 (62.9%) | 1048 (82.6%) |
| Diabetes, n (%) | 23 (1.6%) | 46 (3.1%) | 234 (16.0%) | 7 (0.8%) | 31 (3.6%) | 188 (22.1%) | 55 (4.3%) | 125 (9.6%) | 466 (36.8%) |
| BP-lowering medication, n (%) | 53 (3.6%) | 177 (11.8%) | 378 (25.9%) | 108 (12.7%) | 216 (24.7%) | 284 (33.5%) | 345 (27.2%) | 597 (45.7%) | 742 (58.5%) |
| Lipid-lowering medication, n (%) | 29 (2.0%) | 42 (2.8%) | 87 (6.0%) | 84 (9.9%) | 146 (16.7%) | 214 (25.2%) | 332 (26.2%) | 426 (32.6%) | 518 (40.9%) |
| Glucose-lowering medication, n (%) | 6 (0.4%) | 18 (1.2%) | 105 (7.2%) | 4 (0.5%) | 17 (1.9%) | 95 (11.2%) | 34 (2.7%) | 85 (6.5%) | 329 (25.9%) |
| Metabolic biomarkers, mean (SD) |  |  |  |  |  |  |  |  |  |
| SBP (mmHg) | 115.4 (14.4) | 127.6 (16.4) | 141.2 (20.4) | 118.7 (10.7) | 131.8 (11.9) | 144.4 (14.7) | 115.4 (13.5) | 129.3 (13.8) | 141.6 (18.1) |
| HbA1c (%) | 5.0 (0.4) | 5.1 (0.5) | 5.6 (1.1) | 5.6 (0.3) | 5.7 (0.3) | 6.1 (0.8) | 5.4 (0.4) | 5.6 (0.5) | 6.3 (1.2) |
| non-HDL-C (mmol/L) | 3.4 (0.9) | 3.7 (1.0) | 3.9 (1.0) | 4.1 (1.1) | 4.2 (1.1) | 4.1 (1.2) | 3.9 (0.9) | 4.0 (0.9) | 4.0 (1.0) |
| BMI (kg/m^2^) | 22.9 (12.5) | 23.4 (3.7) | 24.5 (3.9) | 26.6 (4.3) | 27.9 (4.4) | 29.5 (5.2) | 28.1 (5.4) | 29.1 (5.2) | 30.4 (5.5) |

BMI, body mass index; BP, blood pressure; CHARLS, China Health and Retirement Longitudinal Study; ELSA, English Longitudinal Study of Ageing; HbA1c, hemoglobin A1c; HRS, Health and Retirement Study; HSI, hemoglobin A1c-systolic blood pressure index; non-HDL-C, non-high density lipoprotein cholesterol; SBP, systolic blood pressure.

## **Table S2. Associations between the individual and combination of HbA1c and SBP and incident cardiovascular disease.**

| **Predictors/Groups** | **CHARLS** | | **ELSA** | | **HRS** | |
| --- | --- | --- | --- | --- | --- | --- |
|  | **No. of  events/total** | **HR (95% CI)** | **No. of  events/total** | **HR (95% CI)** | **No. of  events/total** | **HR (95% CI)** |
| HbA1c | 1760/6822 | 1.08 (1.03-1.13) | 937/3640 | 1.05 (0.98-1.13) | 1760/6822 | 1.06 (1.02-1.11) |
| SBP |  | 1.16 (1.10-1.22) |  | 1.12 (1.05-1.20) |  | 1.13 (1.08-1.18) |
| HbA1c<6.5&SBP<140 | 1101/4863 | 1.00 [Reference] | 522/2380 | 1.00 [Reference] | 1278/3705 | 1.00 [Reference] |
| HbA1c<6.5&SBP≥140 | 552/1686 | 1.26 (1.13-1.41) | 304/972 | 1.13 (0.98-1.31) | 676/1354 | 1.20 (1.09-1.32) |
| HbA1c≥6.5&SBP<140 | 60/181 | 1.22 (0.91-1.61) | 65/185 | 1.16 (0.85-1.59) | 193/416 | 1.05 (0.88-1.24) |
| HbA1c≥6.5&SBP≥140 | 47/92 | 1.77 (1.29-2.42) | 46/103 | 1.46 (1.05-2.03) | 135/234 | 1.27 (1.05-1.54) |

Cox proportional hazards regression, adjusted for age, sex, and ethnicity, education, socioeconomic status, smoking status, alcohol consumption, non-high density lipoprotein cholesterol, body mass index, and current medications for hypertension, cholesterol, and diabetes, was used to assess the associations. CHARLS, China Health and Retirement Longitudinal Study; CI, confidence interval; ELSA, English Longitudinal Study of Ageing; HbA1c, hemoglobin A1c; HR, hazard ratio; HRS, Health and Retirement Study; SBP, systolic blood pressure.

## **Table S3. Associations between baseline HSI levels and incident cardiovascular disease across diverse subgroups.**

| **Subgroups** | **CHARLS** | | **ELSA** | | **HRS** | |
| --- | --- | --- | --- | --- | --- | --- |
|  | **HR per 1 SD increase (95% CI)** | **P for  interaction** | **HR per 1 SD increase (95% CI)** | **P for  interaction** | **HR per 1 SD increase (95% CI)** | **P for  interaction** |
| Age |  |  |  |  |  |  |
| <60 years | 1.19 (1.12-1.26) | 0.002 | 1.21 (1.04-1.40) | 0.971 | 1.18 (1.08-1.30) | 0.302 |
| ≥60 years | 1.14 (1.07-1.22) |  | 1.12 (1.04-1.21) |  | 1.13 (1.08-1.18) |  |
| Sex |  |  |  |  |  |  |
| Women | 1.17 (1.10-1.24) | 0.795 | 1.16 (1.06-1.27) | 0.703 | 1.14 (1.09-1.21) | 0.669 |
| Men | 1.18 (1.10-1.26) |  | 1.11 (1.00-1.22) |  | 1.14 (1.07-1.22) |  |
| Ethnicity |  |  |  |  |  |  |
| White | NA | NA | 1.14 (1.07-1.22) | 0.611 | 1.13 (1.08-1.18) | 0.967 |
| Non-white | 1.16 (1.11-1.22) |  | 0.97 (0.51-1.85) |  | 1.21 (1.08-1.34) |  |
| High school education |  |  |  |  |  |  |
| Yes | 1.17 (1.02-1.36) | 0.972 | 1.13 (1.04-1.22) | 0.914 | 1.17 (1.05-1.30) | 0.764 |
| No | 1.16 (1.11-1.22) |  | 1.13 (1.01-1.27) |  | 1.13 (1.08-1.19) |  |
| Socioeconomic deprivation |  |  |  |  |  |  |
| Yes | 1.18 (1.05-1.34) | 0.583 | 1.11 (0.95-1.30) | 0.58 | 1.13 (1.07-1.20) | 0.288 |
| No | 1.16 (1.11-1.22) |  | 1.13 (1.05-1.22) |  | 1.16 (1.09-1.24) |  |
| Smoking |  |  |  |  |  |  |
| Yes | 1.23 (1.14-1.34) | 0.736 | 1.22 (1.02-1.45) | 0.57 | 1.21 (1.09-1.36) | 0.599 |
| No | 1.14 (1.08-1.20) |  | 1.12 (1.04-1.21) |  | 1.13 (1.08-1.19) |  |
| Alcohol intake |  |  |  |  |  |  |
| Yes | 1.18 (1.08-1.28) | 0.726 | 1.12 (1.05-1.20) | 0.554 | 1.06 (0.99-1.15) | 0.48 |
| No | 1.16 (1.10-1.22) |  | 1.34 (1.03-1.75) |  | 1.18 (1.12-1.24) |  |
| Hypertension |  |  |  |  |  |  |
| Yes | 1.11 (1.04-1.18) | 0.251 | 1.12 (1.03-1.22) | 0.714 | 1.13 (1.08-1.19) | 0.199 |
| No | 1.12 (1.05-1.19) |  | 1.07 (0.96-1.20) |  | 1.10 (1.01-1.21) |  |
| Diabetes |  |  |  |  |  |  |
| Yes | 1.16 (1.10-1.22) | 0.034 | 1.18 (0.99-1.40) | 0.912 | 1.15 (1.06-1.26) | 0.106 |
| No | 1.18 (1.02-1.37) |  | 1.12 (1.04-1.20) |  | 1.12 (1.07-1.18) |  |
| Body mass index |  |  |  |  |  |  |
| <25 kg/m2 | 1.15 (1.09-1.22) | 0.353 | 1.19 (1.03-1.37) | 0.946 | 1.09 (1.00-1.20) | 0.362 |
| ≥25 kg/m2 | 1.13 (1.04-1.21) |  | 1.12 (1.04-1.21) |  | 1.15 (1.10-1.21) |  |

Cox proportional hazards regression, adjusted for age, sex, and ethnicity, education, socioeconomic status, smoking status, alcohol consumption, non-high density lipoprotein cholesterol, body mass index, and current medications for hypertension, cholesterol, and diabetes, was used to assess the associations. The multiplicative interaction between HSI and stratified variable was examined. CHARLS, China Health and Retirement Longitudinal Study; CI, confidence interval; ELSA, English Longitudinal Study of Ageing; HR, hazard ratio; HRS, Health and Retirement Study; HSI, hemoglobin A1c-systolic blood pressure index.

## **Table S4. Associations between cumulative HSI levels and incident cardiovascular disease across diverse subgroups.**

| **Subgroups** | **CHARLS** | | **ELSA** | | **HRS** | |
| --- | --- | --- | --- | --- | --- | --- |
|  | **HR per 1 SD increase (95% CI)** | **P for  interaction** | **HR per 1 SD increase (95% CI)** | **P for  interaction** | **HR per 1 SD increase (95% CI)** | **P for  interaction** |
| Age |  |  |  |  |  |  |
| <60 years | 1.17 (1.08-1.27) | 0.091 | 1.12 (0.92-1.37) | 0.524 | 1.18 (1.02-1.36) | 0.487 |
| ≥60 years | 1.20 (1.10-1.31) |  | 1.14 (1.02-1.28) |  | 1.15 (1.08-1.23) |  |
| Sex |  |  |  |  |  |  |
| Women | 1.22 (1.13-1.31) | 0.506 | 1.20 (1.04-1.38) | 0.338 | 1.15 (1.06-1.25) | 0.777 |
| Men | 1.18 (1.07-1.31) |  | 1.10 (0.96-1.26) |  | 1.15 (1.04-1.27) |  |
| Ethnicity |  |  |  |  |  |  |
| White | NA | NA | 1.15 (1.04-1.26) | 0.815 | 1.15 (1.07-1.22) | 0.414 |
| Non-white | 1.17 (1.13-1.22) |  | 0.84 (0.23-2.99) |  | 1.17 (0.99-1.38) |  |
| High school education |  |  |  |  |  |  |
| Yes | 1.30 (1.01-1.68) | 0.639 | 1.16 (1.03-1.30) | 0.286 | 1.15 (1.08-1.23) | 0.234 |
| No | 1.18 (1.11-1.26) |  | 1.08 (0.91-1.29) |  | 1.13 (0.96-1.33) |  |
| Socioeconomic deprivation |  |  |  |  |  |  |
| Yes | 1.30 (1.09-1.55) | 0.991 | 1.15 (0.92-1.42) | 0.737 | 1.12 (1.03-1.22) | 0.292 |
| No | 1.18 (1.10-1.26) |  | 1.14 (1.02-1.27) |  | 1.20 (1.09-1.31) |  |
| Smoking |  |  |  |  |  |  |
| Yes | 1.26 (1.13-1.40) | 0.647 | 0.93 (0.70-1.25) | 0.165 | 1.23 (1.02-1.48) | 0.761 |
| No | 1.16 (1.08-1.24) |  | 1.18 (1.06-1.30) |  | 1.14 (1.07-1.22) |  |
| Alcohol intake |  |  |  |  |  |  |
| Yes | 1.11 (0.98-1.27) | 0.647 | 1.14 (1.03-1.26) | 0.739 | 1.14 (1.03-1.25) | 0.351 |
| No | 1.21 (1.13-1.30) |  | 1.47 (1.03-2.10) |  | 1.16 (1.07-1.25) |  |
| Hypertenion |  |  |  |  |  |  |
| Yes | 1.14 (1.05-1.25) | 0.543 | 1.12 (0.99-1.27) | 0.693 | 1.14 (1.05-1.22) | 0.576 |
| No | 1.18 (1.09-1.29) |  | 1.11 (0.95-1.30) |  | 1.12 (0.99-1.26) |  |
| Diabetes |  |  |  |  |  |  |
| Yes | 0.98 (0.80-1.20) | <0.001 | 1.55 (1.21-1.99) | 0.027 | 1.06 (0.92-1.21) | 0.011 |
| No | 1.24 (1.16-1.31) |  | 1.09 (0.98-1.20) |  | 1.18 (1.10-1.27) |  |
| Body mass index |  |  |  |  |  |  |
| <25 kg/m2 | 1.19 (1.10-1.29) | 0.3 | 1.16 (0.94-1.44) | 0.349 | 1.17 (1.03-1.34) | 0.714 |
| ≥25 kg/m2 | 1.15 (1.04-1.28) |  | 1.15 (1.03-1.28) |  | 1.15 (1.08-1.24) |  |

Cox proportional hazards regression, adjusted for age, sex, and ethnicity, education, socioeconomic status, smoking status, alcohol consumption, non-high density lipoprotein cholesterol, body mass index, and current medications for hypertension, cholesterol, and diabetes, was used to assess the associations. The multiplicative interaction between HSI and stratified variable was examined. CHARLS, China Health and Retirement Longitudinal Study; CI, confidence interval; ELSA, English Longitudinal Study of Ageing; HR, hazard ratio; HRS, Health and Retirement Study; HSI, hemoglobin A1c-systolic blood pressure index.

## **Table S5. Associations between baseline and cumulative HSI levels and incident cardiovascular disease after excluding participants who developed CVD within the first two years of follow-up.**

| **Cohorts** | **HR per 1 SD increase (95% CI)** | ***P* value** |  |
| --- | --- | --- | --- |
|  |  |  |  |
| Baseline HSI |  |  |  |
| CHARLS | 1.16 (1.11-1.21) | <0.001 |  |
| ELSA | 1.14 (1.06-1.22) | <0.001 |  |
| HRS | 1.15 (1.10-1.20) | <0.001 |  |
| Cumulative HSI |  |  |  |
| CHARLS | 1.19 (1.12-1.26) | <0.001 |  |
| ELSA | 1.17 (1.06-1.29) | 0.003 |  |
| HRS | 1.15 (1.07-1.23) | <0.001 |  |

Cox proportional hazards regression, adjusted for age, sex, and ethnicity, education, socioeconomic status, smoking status, alcohol consumption, non-high density lipoprotein cholesterol, body mass index, and current medications for hypertension, cholesterol, and diabetes, was used to assess the associations after excluding participants who developed CVD within the first two years of follow-up. CHARLS, China Health and Retirement Longitudinal Study; CI, confidence interval; ELSA, English Longitudinal Study of Ageing; HR, hazard ratio; HRS, Health and Retirement Study; HSI, hemoglobin A1c-systolic blood pressure index.

## **Table S6. Associations between baseline and cumulative HSI levels and incident cardiovascular disease using Fine-Gray regression.**

| **Cohorts** | **HR per 1 SD increase (95% CI)** | ***P* value** |  |
| --- | --- | --- | --- |
|  |  |  |  |
| Baseline HSI |  |  |  |
| CHARLS | 1.16 (1.11-1.21) | <0.001 |  |
| ELSA | 1.13 (1.06-1.21) | <0.001 |  |
| HRS | 1.14 (1.10-1.19) | <0.001 |  |
| Cumulative HSI |  |  |  |
| CHARLS | 1.19 (1.12-1.26) | <0.001 |  |
| ELSA | 1.14 (1.04-1.26) | 0.007 |  |
| HRS | 1.15 (1.08-1.22) | <0.001 |  |

Fine-Gray regression, adjusted for age, sex, and ethnicity, education, socioeconomic status, smoking status, alcohol consumption, non-high density lipoprotein cholesterol, body mass index, and current medications for hypertension, cholesterol, and diabetes, was used to assess the associations. CHARLS, China Health and Retirement Longitudinal Study; CI, confidence interval; ELSA, English Longitudinal Study of Ageing; HR, hazard ratio; HRS, Health and Retirement Study; HSI, hemoglobin A1c-systolic blood pressure index.

## **Table S7. Associations between baseline and cumulative HSI levels and incident cardiovascular disease using modified Poisson regression.**

| **Cohorts** | **RR per 1 SD increase (95% CI)** | ***P* value** |  |
| --- | --- | --- | --- |
|  |  |  |  |
| Baseline HSI |  |  |  |
| CHARLS | 1.13 (1.09-1.17) | <0.001 |  |
| ELSA | 1.11 (1.05-1.16) | <0.001 |  |
| HRS | 1.09 (1.06-1.12) | <0.001 |  |
| Cumulative HSI |  |  |  |
| CHARLS | 1.15 (1.10-1.21) | <0.001 |  |
| ELSA | 1.12 (1.03-1.21) | 0.01 |  |
| HRS | 1.10 (1.05-1.15) | <0.001 |  |

Modified Poisson regression, adjusted for age, sex, and ethnicity, education, socioeconomic status, smoking status, alcohol consumption, non-high density lipoprotein cholesterol, body mass index, and current medications for hypertension, cholesterol, and diabetes, was used to assess the associations. CHARLS, China Health and Retirement Longitudinal Study; CI, confidence interval; ELSA, English Longitudinal Study of Ageing; HR, hazard ratio; HRS, Health and Retirement Study; HSI, hemoglobin A1c-systolic blood pressure index.

**
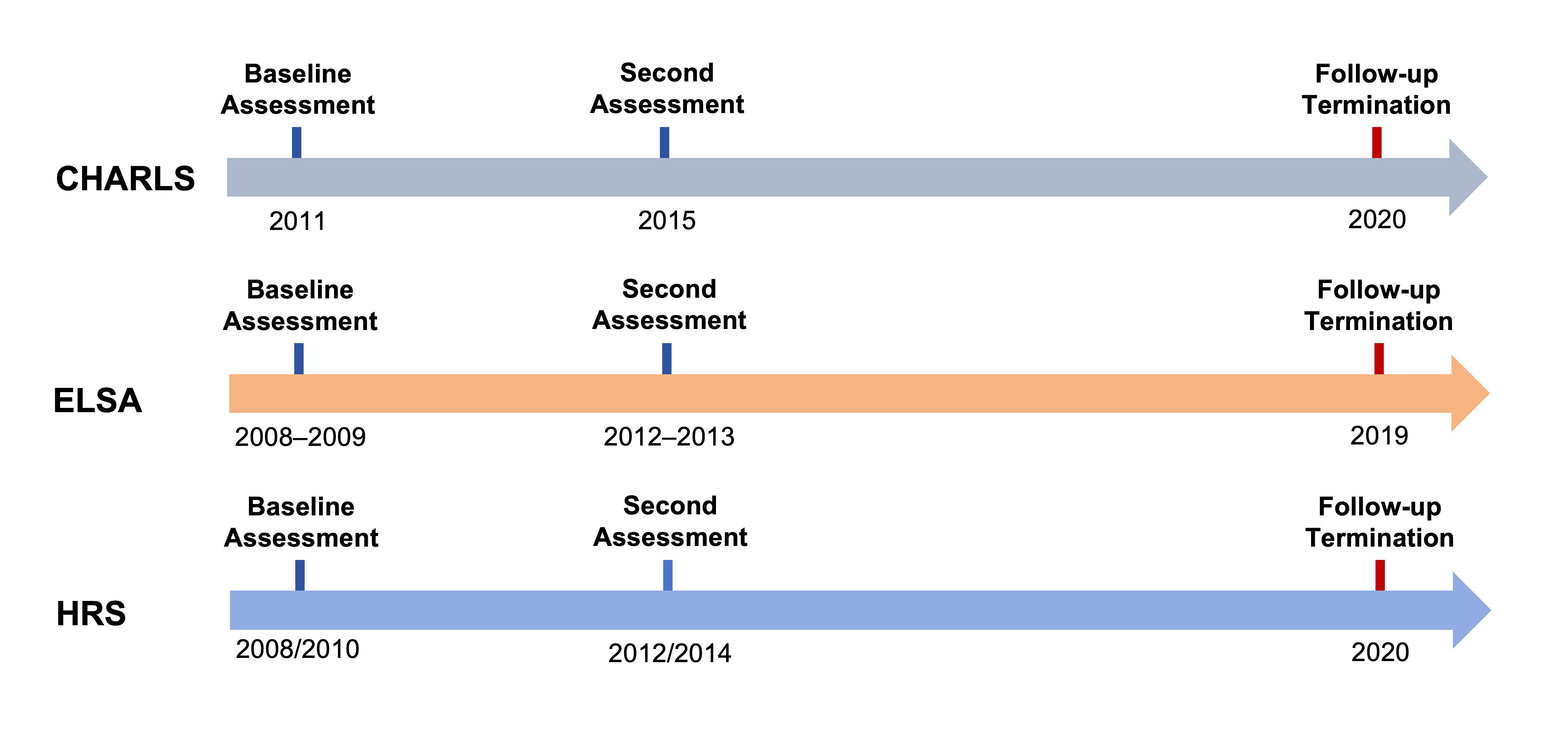
**

## **Figure S1. Timelines of study design.**

CHARLS, China Health and Retirement Longitudinal Study; ELSA, English Longitudinal Study of Ageing; HRS, Health and Retirement Study.

**
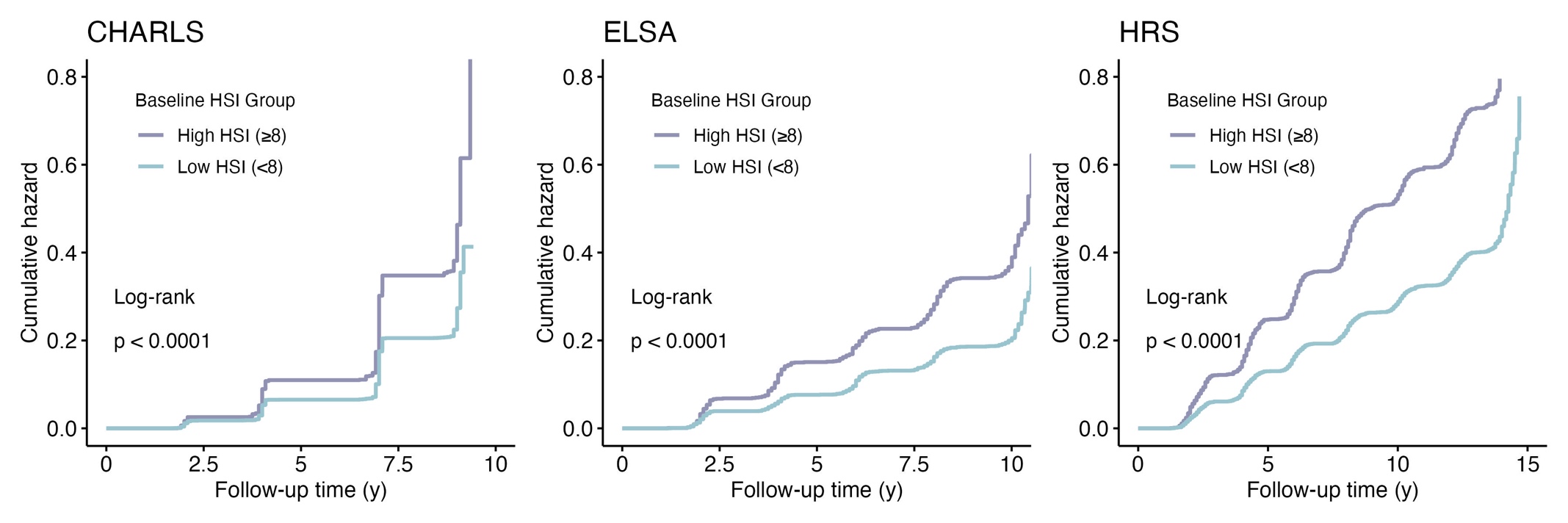
**

## **Figure S2. Cumulative hazard curve plots of incident cardiovascular disease according to the optimal cutpoint of baseline HSI.**

CHARLS, China Health and Retirement Longitudinal Study; ELSA, English Longitudinal Study of Ageing; HR, hazard ratio; HRS, Health and Retirement Study.

**
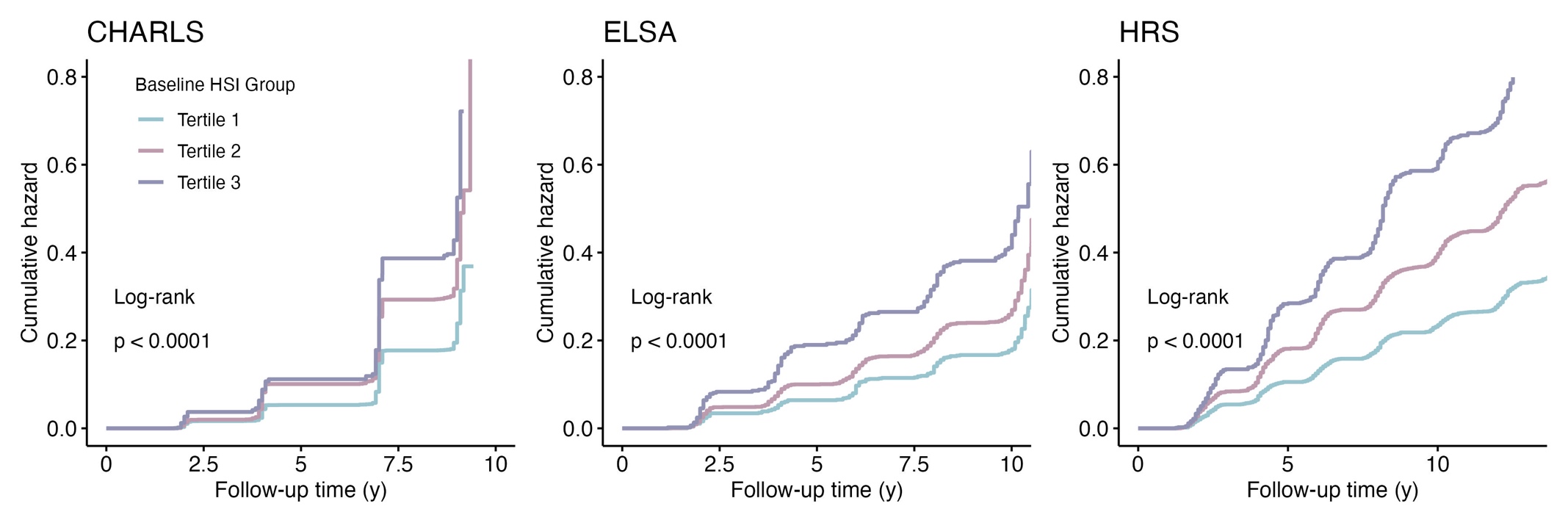
**

## **Figure S3. Cumulative hazard curve plots of incident cardiovascular disease according to the clinical cutpoints of baseline HSI.**

CHARLS, China Health and Retirement Longitudinal Study; ELSA, English Longitudinal Study of Ageing; HR, hazard ratio; HRS, Health and Retirement Study.
